# Intracranial stenting with the Neuroform Atlas Stent for symptomatic intracranial atherosclerotic stenosis: a bi-center retrospective analysis including stroke recurrence nomogram

**DOI:** 10.3389/fneur.2025.1507339

**Published:** 2025-03-25

**Authors:** Weicheng Peng, Haiyang Ma, Xinli Xiang, Rui Zhao, Meng Lv, Sheng Xu, Yuhua Jiang, Zhiqiang Hu, Feng Guan

**Affiliations:** ^1^Department of Neurosurgery, Beijing Shijitan Hospital, Capital Medical University, Beijing, China; ^2^Department of Pharmacy, Beijing Shijitan Hospital, Capital Medical University, Beijing, China; ^3^Department of Neurosurgery, Beijing Neurosurgical Institute, Beijing Tiantan Hospital, Capital Medical University, Beijing, China

**Keywords:** intracranial atherosclerotic stenosis, ischemic stroke, intracranial stenting, Neuroform Atlas Stent, predictive nomogram

## Abstract

**Background:**

Intracranial stenting with the Neuroform Atlas Stent is an emerging option for patients with symptomatic intracranial atherosclerotic stenosis (sICAS) who do not respond to intensive medical treatment. However, the efficacy, safety, and risk factors associated with postoperative stroke recurrence remain controversial.

**Methods:**

A total of 326 consecutive patients with sICAS treated with intracranial stenting using the Neuroform Atlas Stent were retrospectively analyzed to evaluate the efficacy and safety of the procedure. Patients were randomly assigned to a training set and a validation set in a 7:3 ratio. Significant variables in the univariate logistic analyses were included in the final multivariate logistic regression analyses in the training set. Subsequently, we developed a predictive nomogram for sICAS treated with a Neuroform Atlas Stent to predict the likelihood of stroke recurrence at 6 months.

**Results:**

The overall mean stenosis rate of the target artery was 88.85% ± 6.53% before the stenting (T0), 47.58% ± 9.94% at the end of the procedure (T1), and 40.21% ± 7.77% at the 6-month follow-up (T2). The stenosis rate was statistically significant between T0 and T1 (*p* < 0.01) and between T0 and T2 (*p* < 0.01). At 6 months postoperatively, 36 patients had a stroke recurrence linked to the target artery. Diabetes, acute ischemic stroke (AIS), plaque burden on vessel wall MRI, enhancement ratio on vessel wall MRI, and stenosis (T1) were independent predictors of stroke recurrence. A predictive nomogram was developed, showing strong predictive capability with the area under the curve of 0.933 for the training set and 0.949 for the validation set.

**Conclusion:**

Intracranial stenting with the Neuroform Atlas Stent is a potentially safe and effective treatment for sICAS. Risk factors for recurrent stroke post-procedure include diabetes, current smoker, current drinker, AIS, plaque burden, enhancement ratio, and stenosis (T1).

## 1 Introduction

Stroke is the second leading cause of death globally and the top cause of mortality and disability in China (1, 2), with over 2 million new cases annually. It is the primary cause of death in 34 provinces, with ischemic stroke (IS) being the predominant type. Stroke results in the highest loss of disability-adjusted life years among all diseases (3, 4). As the most common types of cerebrovascular events in China, IS and transient ischemic attack (TIA) account for approximately 70% of all strokes (5). The burden of stroke is anticipated to rise due to an aging population, a high prevalence of risk factors (such as hypertension), and limited treatment options. The age-standardized incidence of acute ischemic stroke (AIS) ranges from 97.1 to 127.3 per 100,000 person-years, with a mortality-to-incidence ratio of 0.26 to 0.29 (6). Intracranial atherosclerotic stenosis (ICAS) is a significant contributor to AIS.

Recent studies have indicated that 31% of elderly individuals with common cardiovascular risk factors have ICAS, with 9% of these patients having stenosis greater than 50%. Suri et al. found that 4.2% of ICAS patients had 50–69% stenosis, 3.1% had 70–99, and 1.7% were completely occluded (7). Recent studies from the WEAVE (Wingspan Stent System Post Market Surveillance)/WOVEN (Wingspan One-year Vascular Events and Neurologic Outcomes) trial have demonstrated that intracranial stenting is an effective intervention for preventing stroke recurrence in patients with symptomatic ICAS (sICAS) who do not respond to intensive medical treatment (8, 9). Intracranial stenting with the Neuroform Atlas Stent is emerging as a promising option for these patients (10, 11).

Intracranial stenting is subject to stringent patient selection criteria due to the potential for perforator occlusion, which is a significant mechanism behind stroke recurrence. Furthermore, endovascular treatment may not provide any advantages and could introduce unnecessary risks of perioperative complications (12). The rates of stroke recurrence or death observed during follow-up periods are significant, with rates of 2.6% in the WEAVE study (72-h periprocedural) (8) and 14.7% in Stenting and Aggressive Medical Management for the Preventing Recurrent Stroke in Intracranial Stenosis (SAMMPRIS, 30-day follow-up) (13). Although the association between risk factors and stroke recurrence has been studied extensively, uncertainties remain. Previous studies have reported statistically significant correlations between stroke recurrence in sICAS and several risk factors, including watershed infarct pattern, site of stenosis, intracranial atherosclerotic burden, elevated low-density lipoprotein cholesterol levels, elevated systolic blood pressure, older age, and black race (14, 15, 16). Conversely, factors such as smoking and body mass index showed no correlation, whereas high-density lipoprotein levels and the usage of cholesterol-lowering medications were found to be protective against sICAS (7). However, studies exploring the relationship between stroke recurrence and risk factors in sICAS patients after intracranial stenting are limited. The incidence of stroke recurrence after intracranial stenting and the risk factors for postoperative stroke recurrence remain controversial.

The present bi-center study initially validated the efficacy and safety of intracranial stenting utilizing the Neuroform Atlas Stent in a cohort of 326 patients with sICAS. However, comprehensive studies on stroke recurrence and risk factors are essential for predicting postoperative stroke recurrence and identifying patients suitable for intracranial stenting with the Neuroform Atlas Stent. The current bi-center, retrospective, observational study sought to evaluate the efficacy and safety of intracranial stenting with the Neuroform Atlas Stent for treating sICAS and develop a predictive model for stroke recurrence at 6 months postoperatively based on independent risk factors.

## 2 Materials and methods

This bi-center, retrospective study was conducted in accordance with the tenets of the Helsinki Declaration of 1975 as revised in 2000. The study was approved by the Institutional Review Board of Beijing Shijitan Hospital, Capital Medical University (Approval No.sjtkyll-lx-2022-52). All patients or their legal guardians in this study provided informed consent.

### 2.1 Patient recruitment

The inclusion criteria were as follows: (1) Patients aged >18 years. (2) ICAS (stenosis rate > 70 and < 100%) was confirmed preoperatively in single-vessel [M1 segment of middle cerebral artery (MCA), basilar artery (BA), or intracranial segment of vertebral artery (VA)]. (3) Patients with recurrent IS or TIA within the past 90 days due to hypoperfusion in the territory of the target lesion despite receiving intensive medical treatment, where IS was defined as a new focal neurological deficit lasting ≥24 h or < 24 h with new infarction on imaging, and TIA was defined as acute onset of focal neurological deficit lasting <24 h without new infarction on imaging. (4) Patients with significant hypoperfusion manifestations in the brain tissue innervated by the target artery, as evidenced by computed tomography perfusion (CTP), showing a cerebral blood flow decrease of ≥30% compared with the perfusion on the contralateral side for an anterior circulation lesion, or the anterior circulation territory for a posterior circulation lesion (17). (5) All patients underwent intracranial stenting with the Neuroform Atlas Stent, performed by the same surgical team from the Beijing Shijitan Hospital or Beijing Tiantan Hospital.

The exclusion criteria were as follows: (1) Patients with massive stroke in the past 30 days. (2) Those with a side branch neoanastomotic vessel and no hypoperfusion on CTP. (3) Those presenting with cerebral infarction combined with cerebral hemorrhage. (4) Non-atherosclerotic stenosis, including moyamoya disease, muscle fiber dysplasia, or arterial dissection. (5) Patients with severe illnesses who could not tolerate anesthesia and surgery.

### 2.2 Clinical data

**Figure 1:**
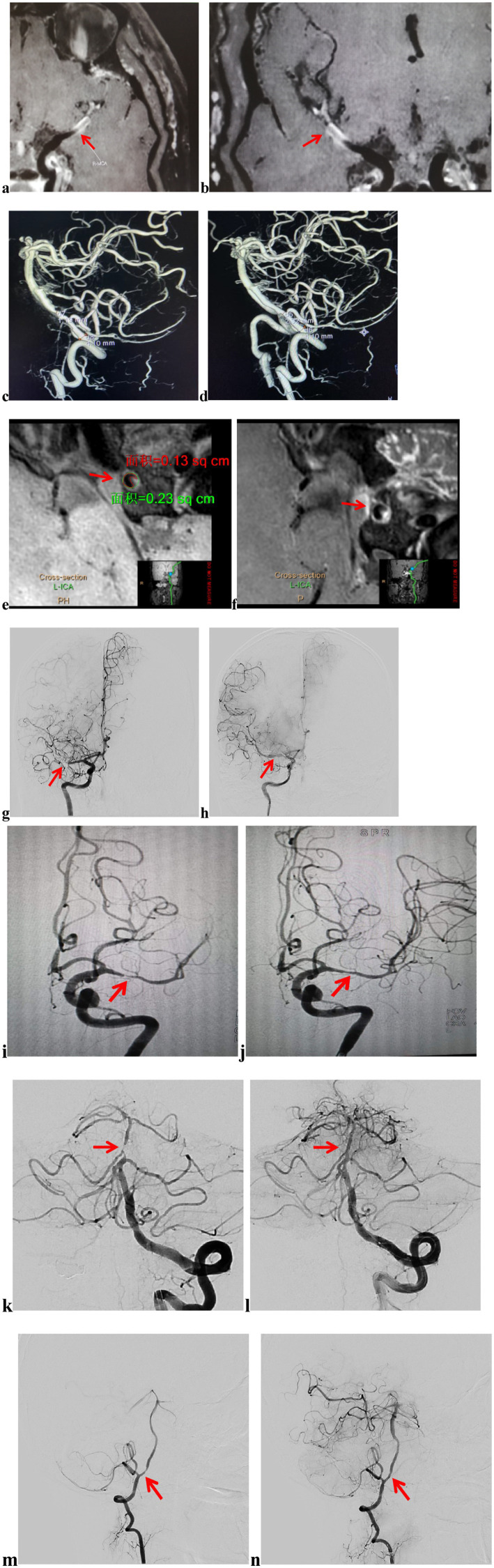
Imaging data and endovascular procedure. Preoperative VW-HRMRI showed that the lumen of M1 of the right MCA was almost occluded, but a portion of the true lumen of the vessel wall remained, making it a candidate for angioplasty and stenting **(a,b)**. The stenosis rate = 1-(Ds/Dn), where Ds was the diameter of the artery where the stenosis was most obvious and Dn was the diameter of the proximal normal artery. The stenosis rate of the left VA was 70.98% before intracranial stenting **(c)**, 43.41% after intracranial stenting with the Neuroform Atlas Stent **(d)**; plaque burden was measured at the site of maximal stenosis, calculated using the formula: (outer area-lumen area)/outer area **(e)**; the contrast enhancement ratio was measured at the site of maximum plaque enhancement, normalizing the SI by using nearby gray matter (**e**, post-contrast SI; **f**, pre-contrast SI); preoperative DSA showed that the lumen of M1 of the right MCA was nearly occluded, and the distal branches were not visualized **(g)**; after angioplasty, a Neuroform Atlas Stent was successfully implanted in the M1 of the right MCA, and follow-up angiography 10 min later showed good blood flow **(h)**; preoperative DSA showed that the lumen of M1 of the left MCA was severe stenosis, and the distal branches were sparse **(i)**; after angioplasty, a Neuroform Atlas Stent was successfully implanted in the M1 of the left MCA, and follow-up angiography 10 min later showed good blood flow **(j)**; preoperative DSA showed that the lumen of BA was severe stenosis, and the distal branches were sparse **(k)**; after angioplasty, a Neuroform Atlas Stent was successfully implanted in the BA, and follow-up angiography 10 min later showed good blood flow **(l)**. Preoperative DSA showed that the lumen of the left VA was severe stenosis, and the distal branches were sparse **(m)**. After angioplasty, a Neuroform Atlas Stent was successfully implanted in the V4 of the left VA, and follow-up angiography 10 min later showed good blood flow **(n)**. VW-HRMRI, vascular wall high-resolution magnetic resonance imaging; MCA, middle cerebral artery; VA, vertebral artery; SI: signal intensity; DSA, digital subtraction angiography; BA, basilar artery.

Upon hospital admission, all patients underwent computed tomography (CT) and magnetic resonance imaging (MRI) (including diffusion-weighted imaging (DWI), T2, T1, and fluid-attenuated inversion recovery (FLAIR) sequences). Thereafter, computed tomography angiography (CTA) and CTP were performed to assess the extent of intracranial and extracranial vascular patency and brain tissue perfusion. The degree of stenosis in the target artery was assessed using digital subtraction angiography (DSA). Radiological features of plaques were extracted from vessel wall high-resolution magnetic resonance imaging (VW-HRMRI) (Figures 1a,b). Follow-up clinical status was assessed by a team of experienced senior neurosurgeons and neuroimaging physicians, who collaboratively evaluated clinical and imaging data. Laboratory assessments included routine blood counts, platelet aggregation tests, thromboelastograms, and genetic testing for clopidogrel resistance. Clinical assessments included physical examination, neurological examination, and the modified Rankin Scale (mRS).

Stenosis rate, plaque burden, and the enhancement ratio were calculated as follows: (1) Stenosis rate = 1-(Ds/Dn), where Ds is the diameter of the artery where the stenosis was most obvious and Dn is the diameter of the proximal normal artery (Figures 1c,d) (18). (2) Plaque burden was measured at the site of maximal stenosis, calculated using the formula: (outer area-lumen area)/outer area (Figure 1e) (19). (3) The contrast enhancement ratio was measured at the site of maximum plaque enhancement, normalizing the signal intensity (SI) by referencing adjacent gray matter (in a region of the hippocampus that is approximately 15 mm^2^) (Figures 1e,f). The contrast enhancement ratio of plaque was calculated using the formula: (post-contrast SI of plaque/post-contrast SI of gray matter)/(pre-contrast SI of plaque/pre-contrast SI of gray matter) (20, 21).

### 2.3 Endovascular procedure

All intracranial stenting procedures with the Neuroform Atlas Stent were performed under general anesthesia at either Beijing Shijitan Hospital or Beijing Tiantan Hospital by two senior neurointerventionalists, each possessing over a decade of expertise in intracranial stenting. During the operation, the anesthetist maintained the patient’s systolic pressure between 90 and 140 mmHg, monitored through continuous arterial line pressures, to mitigate significant variations in blood pressure and heart rate. Each patient had a working channel established in the right femoral artery via the Seldinger technique, followed by cerebral angiography with a 5F contrast catheter to assess the affected vessel and stenosis rate of the target artery. Intracranial stenting was performed using a triaxial technique with a 6F long sheath (Neuron Max 088 Penumbra, USA) and a Navien 6F 0.072-inch 105-cm ID distal intracranial catheter (Covidien Vascular Therapies). Patients received heparinization with 3,000 IU of heparin sodium in saline, targeting a peak-activated clotting time of 200–300 s, ideally 250 s (22).

In patients with sICAS of the MCA, the intraoperative access catheter was specifically directed to the distal C1 segment of the ipsilateral internal carotid artery, while the intermediate catheter was advanced coaxially to the C4 segment. A microguidewire facilitated the careful advancement of the microcatheter through the stenotic segment of the MCA, allowing for the coaxial delivery of the microcatheter across the stenotic segment. Intraoperative microcatheterography confirmed that the microcatheter was situated within the true lumen, leading to the replacement of the long microcatheter wire. This wire enabled the coaxial delivery of the Gateway balloon to the stenotic segment, where it was gradually inflated the balloon to 6 atmospheres and maintained for 15 s before being deflated. Imaging results indicated dilation of the stenosis, roughness of the internal wall, and a measurable residual stenosis rate. After a 10-min observation and subsequent re-imaging, residual stenosis was noted in the stenotic segment. The microcatheter was then advanced coaxially along the microguidewire to the distal end of the stenotic segment. The Neuroform Atlas Stent was deployed against the medial wall of the stenotic segment, achieving satisfactory expansion and apposition. Final imaging revealed the presence of residual stenosis. All patients underwent suturing at the puncture site using an Abbott Vascular Suture Device, with hemostasis achieved through freehand and sandbag compression. Selected imaging data and details of the endovascular procedure are illustrated in Figure 1.

### 2.4 Postprocedural evaluation and medical therapy

Before the intracranial stenting procedure, each patient received dual antiplatelet medication with aspirin (100 mg once daily) and clopidogrel (75 mg once daily). For those identified as hyporesponders to clopidogrel, an additional regimen of ticagrelor (90 mg twice daily) was administered. Following the stenting procedure, each patient underwent DSA to confirm the morphological stability of the Neuroform Atlas Stent. Postoperatively, all patients were closely monitored in the intensive care unit (ICU) until they were transferred to the general ward after the removal of dressings. Subsequently, patients then received optimal pharmacological treatment based on their comorbidities. After the intracranial stenting, patients were prescribed either aspirin (100 mg once daily) plus clopidogrel (75 mg once daily) or ticagrelor (90 mg twice daily). In particular, clopidogrel or ticagrelor was discontinued 90 days post-stenting, contingent upon the results of the antiplatelet drug resistance test. Platelet function assessments, including the platelet aggregation test, were performed for all patients before and after the stenting procedure.

### 2.5 Radiological and clinical follow-up

Patients underwent a neurological examination (including assessment of mRS). The stenosis rate of the ICAS was measured using DSA before and immediately following the surgical procedure. The two assessment periods are designated as “T0” and “T1,” respectively. All patients were scheduled for follow-up DSA and CTA 6 months postoperatively, referred to as “T2.” The entire canal lumen was evaluated, and the stenosis rate of ICAS at each time point was determined by three experienced neurointerventionalists, who calculated the average of the ICAS measurements at each time interval.

A neurologist with over 5 years of experience confirmed a recurrent stroke linked to the target artery. The diagnostic criteria included the following: (1) new focal neurological deficits in the previously affected area lasting over 24 h, supported by imaging evidence; (2) more than two TIA symptoms in the region of the ICAS, even without clear imaging evidence of stroke; and (3) ruling out other causes such as cerebral hemorrhage or tumors (23).

### 2.6 Statistical analyses

Statistical analyses were conducted using SPSS 25.0 software and R software version 4.1.1. The chi-square test or Fisher’s exact test was used for the analysis of dichotomous variables. Patients were randomly assigned a training set and a validation set in a 7:3 ratio. Variables that demonstrated significance in the univariate logistic analyses were incorporated into the final multivariate logistic regression analyses in the training set. A two-sided test was performed with a significance level set at *α* = 0.05.

## 3 Results

### 3.1 Baseline characteristics

A total of 358 consecutive patients with sICAS who received intracranial stenting with the Neuroform Atlas Stent at Beijing Shijitan Hospital and Beijing Tiantan Hospital between June 2020 and September 2023 were evaluated. Out of these, 326 patients (mean age 60.52 ± 9.73 years, 61.66% men) met the criteria for inclusion in our study, and all 326 (100.0%) were monitored through DSA. Among the 32 patients excluded from the study, 27 did not return for DSA assessment, and 5 succumbed to other systemic diseases within 6 months following the stenting procedure; none of the patients experienced mortality related to intracranial stenting or IS. Of the 326 patients, 136 had sICAS of the MCA, 105 of the BA, and 85 of the VA. Regarding the qualifying ischemic event, 229 patients had AIS (229/326, 70.25%) and 97 had TIA (97/326, 29.75%). Baseline characteristics of the study cohort are displayed in Table 1.

**Table 1:** Baseline characteristics of patients (*n* = 326).

Variables	Values (*n* = 326)
Mean age ± SD, yrs	60.52 ± 9.73
Males, *n* (%)	201 (61.66)
Hypertension	247 (75.77)
Dyslipidemia	269 (82.52)
Diabetes	194 (59.51)
Cardiovascular disease	190 (58.28)
Smoke exposure, *n* (%)	
Current smoker	127 (38.96)
Previous smoker	46 (14.11)
Never smoked	153 (46.93)
Alcoholism, *n* (%)	
Current drinker	107 (32.82)
Former drinker	32 (9.82)
Never drank	187 (57.36)
Target artery, *n* (%)	
MCA	136 (41.72)
BA	105 (32.21)
VA	85 (26.07)
Qualifying ischemic events, *n* (%)	
AIS	229 (70.25)
TIA	97 (29.75)
Plaque radiological features	
Plaque burden	0.69 ± 0.12
Plaque thickness	1.56 ± 0.58
Enhancement ratio	2.47 ± 1.00

MCA, middle cerebral artery; BA, basilar artery; VA, vertebral artery; AIS, acute ischaemic stroke; TIA, transient ischaemic attacks.

### 3.2 Early and 6-months outcome

The intracranial stenting procedure had a 100% success rate (358/358), with 326 patients completing the 6-month radiological follow-up. Before intracranial stenting, 105 (105/326, 32.21%) patients had mRS scores of 0–2, whereas 221 (221/326, 67.79%) had scores of 3–5. At the 6-month follow-up, 319 (319/326, 97.85%) patients had a favorable prognosis (mRS 0–2), whereas seven (7/326, 2.15%) experienced an unfavorable outcome (mRS 3–5). A paired-sample *t*-test conducted on the preoperative and postoperative mRS scores revealed a statistically significant difference (*p* = 0.027). No patient required repeat endovascular treatment after intracranial stenting.

The mean length of ICAS was 7.70 ± 2.80 mm, with a range of 0.9 mm and 16.1 mm and a median of 7.5 mm. A notable improvement in target vessel caliber was achieved, with the successful restoration of the stenotic lumen compared with preoperative conditions. The mean stenosis rate was 88.85% ± 6.53% before intracranial stenting with the Neuroform Atlas Stent (T0), 47.58% ± 9.94% at the end of treatment (T1), and 40.21% ± 7.77% at 6-month follow-up (T2). The mean reduction in ICAS from T0 to Tl was 41.27% ± 11.81% and from T1 to T2 was 7.37% ± 12.08%. The mean reduction in ICAS from T0 to T2 was 48.64% ± 10.51%. The paired-samples *t*-test showed that the stenosis rate of ICAS was statistically significant between T0 and T1, between T0 and T2, and between T1 and T2 (all *p* < 0.01) (Table 2).

**Table 2:** Procedural details and outcomes (*n* = 326).

Pre/post-treatment data	Values (mean ± sd)	*t*-value	*P*-value
ICAS length (mm)	7.70 ± 2.80	-	-
Stenosis rate			
Stenosis rate T0 (%)	88.85 ± 6.53	-	-
Stenosis rate T1 (%)	47.58 ± 9.94	-	-
Stenosis rate T2 (%)	40.21 ± 7.77	-	-
Percentage reduction			
Percentage reduction T0-T1 (↓%)	41.27 ± 11.81	-	-
Percentage reduction T0-T2 (↓%)	48.64 ± 10.51	-	-
Percentage reduction T1-T2 (↓%)	7.37 ± 12.08	-	-
Percentage of patients with a mRS 0–2			
Pre-stenting	32.21% (105/326)	-	-
Post-stenting	67.79% (221/326)	-	-
Paired-Samples *T* Test			
T0 vs. T1	41.27 ± 11.80	63.11	0.00
T0 vs. T2	48.64 ± 10.51	83.55	0.00
T1 vs. T2	7.37 ± 12.80	11.01	0.00
mRS-pre vs. mRS-post	0.91 ± 0.81	12.52	0.027

ICAS, intracranial atherosclerotic stenosis; mRS, modified Rankin Scale.

There were five cases of ischemic strokes, three cases of hemorrhagic strokes related to the intracranial stenting process, four cases of transient central facial paralysis, eight cases of transient limb numbness, and seven cases of transient dysphagia. No deaths or additional endovascular interventions were required after intracranial stenting. The overall complication rate for the Neuroform Atlas Stent was 8.28% (27/326), with an in-stent restenosis (ISR) rate (stenosis rate ≥ 70%) of 1.84% (6/326) at 6 months post-surgery. In addition, 36 patients had stroke recurrences linked to the target artery, resulting in a recurrence rate of 11.04% (36/326), with no reported mortality.

### 3.3 Comparison of clinical and radiological features

Of the 326 patients included in this study, 201 were males and 125 were females, with 157 aged ≤60 years and 169 aged >60 years. A total of 247 patients had hypertension, 269 had dyslipidemia, 194 had diabetes, and 190 had cardiovascular disease. Smoking status included 127 current smokers, 46 previous smokers, and 153 individuals who had never smoked. In terms of alcohol consumption, 107 were current drinkers, 32 were former drinkers, and 187 had never consumed alcohol. The target vessels included 136 cases of MCA, 105 cases of BA, and 85 cases of VA. The qualifying ischemic events consisted of 229 cases of AIS and 97 cases of TIA. All patients were followed up for >6 months, with an average follow-up duration of 16.5 ± 2.4 months. Thirty-six patients experienced recurrence. Variables such as diabetes, current smokers, current drinkers, AIS, plaque burden, enhancement ratio, and stenosis (T1) showed statistically significant differences between different groups (*p* < 0.05, Table 3). Stroke recurrence occurred in 10.09% (23/228) patients in the training set and 5.70% (13/98) in the validation set.

**Table 3:** Baseline characteristics and comparison of stroke recurrence in sICAS patients treated with intracranial stenting with the Neuroform Atlas Stent (*n* = 326).

Variables	sICAS patients treated with intracranial stenting		*χ*^2^/t value	*P* value
	Recurrence (*n* = 36)	Recurrence-free (*n* = 290)		
Sex, *n* (%)			1.038	0.308
Female	11 (30.56)	114 (39.31)		
Male	25 (69.44)	176 (60.69)		
Age (years), *n* (%)			0.887	0.346
≤ 60	20 (55.56)	137 (47.24)		
> 60	16 (44.44)	153 (52.76)		
Hypertension, *n* (%)			0.013	0.909
Yes	27 (75.00)	220 (75.86)		
No	9 (25.00)	70 (24.14)		
Dyslipidemia, *n* (%)			0.108	0.743
Yes	29 (80.56)	240 (82.76)		
No	7 (19.44)	50 (17.24)		
Diabetes, *n* (%)			14.497	<0.001^*^
Yes	32 (88.89)	162 (55.86)		
No	4 (11.11)	128 (44.14)		
Cardiovascular disease, *n* (%)			0.133	0.715
Yes	22 (61.11)	168 (57.93)		
No	14 (38.89)	122 (42.07)		
Smoke exposure, *n* (%)			8.235	0.016
Current smoker	21 (58.33)	106 (36.55)		
Previous smoker	6 (16.67)	40 (13.79)		
Never smoked	9 (25.00)	144 (49.66)		
Alcoholism, *n* (%)			7.396	0.025
Current drinker	19 (52.78)	88 (30.34)		
Former drinker	2 (5.56)	30 (10.34)		
Never drank	15 (41.66)	172 (59.32)		
Target vessel, *n* (%)			0.079	0.961
MCA	15 (41.67)	121 (41.72)		
BA	11 (30.56)	94 (32.41)		
VA	10 (27.77)	75 (25.87)		
AIS vs. TIA, *n* (%)			11.338	0.001
AIS	34 (94.44)	195 (67.24)		
TIA	2 (5.56)	95 (31.72)		
Plaque radiological features				
Plaque burden	0.81 ± 0.13	0.67 ± 0.11	−6.995	<0.001^*^
Plaque thickness	1.57 ± 0.68	1.56 ± 0.57	−0.141	0.888
Enhancement ratio	3.49 ± 1.09	2.34 ± 0.92	−6.919	<0.001^*^
Stenosis (T0), %	90.33 ± 6.22	88.67 ± 6.56	−1.439	0.151
Stenosis (T1), %	54.33 ± 12.16	46.74 ± 9.32	−4.440	<0.001^*^

sICAS, Symptomatic intracranial atherosclerotic stenosis; *p value<0.001.

### 3.4 Risk factors for stroke recurrence after intracranial stenting with the Neuroform Atlas Stent

Univariate analysis revealed that diabetes [odds ratio (OR) = 5.000, 95% confidence interval (CI): 1.445–17.301], current smoker (OR = 4.192, 95% CI: 1.458–12.048), current drinker (OR = 2.344, 95% CI: 0.975–5.637), symptom of AIS (OR = 11.756, 95% CI: 1.555–88.885), plaque burden on vessel wall MRI (OR = 1.118, 95% CI: 1.068–1.170), enhancement ratio on vessel wall MRI (OR = 3.013, 95% CI: 1.843–4.927), and stenosis (T1) (OR = 1.095 95% CI: 1.042–1.151) were significantly associated with stroke recurrence in patients with ICAS receiving the Neuroform Atlas Stent (*p* < 0.05, Table 4). Multivariate logistic regression analyses showed that diabetes (OR = 5.050, 95% CI: 1.119–22.786), AIS (OR = 13.846, 95% CI: 1.301–147.322), plaque burden on vessel wall MRI (OR = 1.094, 95% CI: 1.035–1.157), enhancement ratio on vessel wall MRI (OR = 3.012, 95% CI: 1.551–5.848), and stenosis (T1) (OR = 1.098, 95% CI: 1.024–1.178) were independent predictors of stroke recurrence (*p* < 0.05, Table 4).

**Table 4:** Univariate analysis and multivariate logistic regression analyses of risk factors for stroke recurrence in training set (*n* = 228).

Variables	Univariate analysis		Multivariate analysis	
	OR (95% CI)	*P* Value	OR (95% CI)	*P* value
Diabetes, *n* (%)				
Yes	5.000 (1.445–17.301)	0.011	5.050 (1.119–22.786)	0.035
No	Reference		Reference	
Smoke exposure, *n* (%)				
Current smoker	4.192 (1.458–12.048)	0.008	2.053 (0.511–8.257)	0.311
Previous smoker	2.814 (0.709–11.162)	0.141		
Never smoked	Reference		Reference	
Alcoholism, *n* (%)				
Current drinker	2.344 (0.975–5.637)	0.057	1.748 (0.518–5.896)	0.368
Former drinker	0.626 (0.076–5.176)	0.664		
Never drank	Reference		Reference	
AIS vs. TIA, *n* (%)				
AIS	11.756 (1.555–88.885)	0.017	13.846 (1.301–147.322)	0.029
TIA	Reference		Reference	
Plaque radiological features				
Plaque burden	1.118 (1.068–1.170)	<0.001^*^	1.094 (1.035-1.157)	0.001
Plaque thickness	1.315 (0.645–2.679)	0.451		
Enhancement ratio	3.013 (1.843–4.927)	<0.001^*^	3.012 (1.551-5.848)	0.001
Stenosis (T0), %	1.027 (0.959–1.099)	0.446		
Stenosis (T1), %	1.095 (1.042–1.151)	<0.001^*^	1.098 (1.024-1.178)	0.008
Sex				
Female	Reference			
Male	2.143 (0.816–5.624)	0.122		
Age (years)				
≤ 60	Reference			
> 60	0.805 (0.345–1.877)	0.616		
Hypertension, *n* (%)				
Yes	Reference			
No	1.815 (0.517–6.375)	0.353		
Dyslipidemia, *n* (%)				
Yes	0.643 (0.238–1.733)	0.382		
No	Reference			
Cardiovascular disease, *n* (%)				
Yes	1.215 (0.508–2.905)	0.662		
No	Reference			
Target vessel, *n* (%)				
MCA	Reference			
BA	1.191 (0.468–3.031)	0.714		
VA	0.589 (0.176–1.974)	0.391		

*P value<0.001.

### 3.5 Development and validation of the predictive model

**Figure 2:**
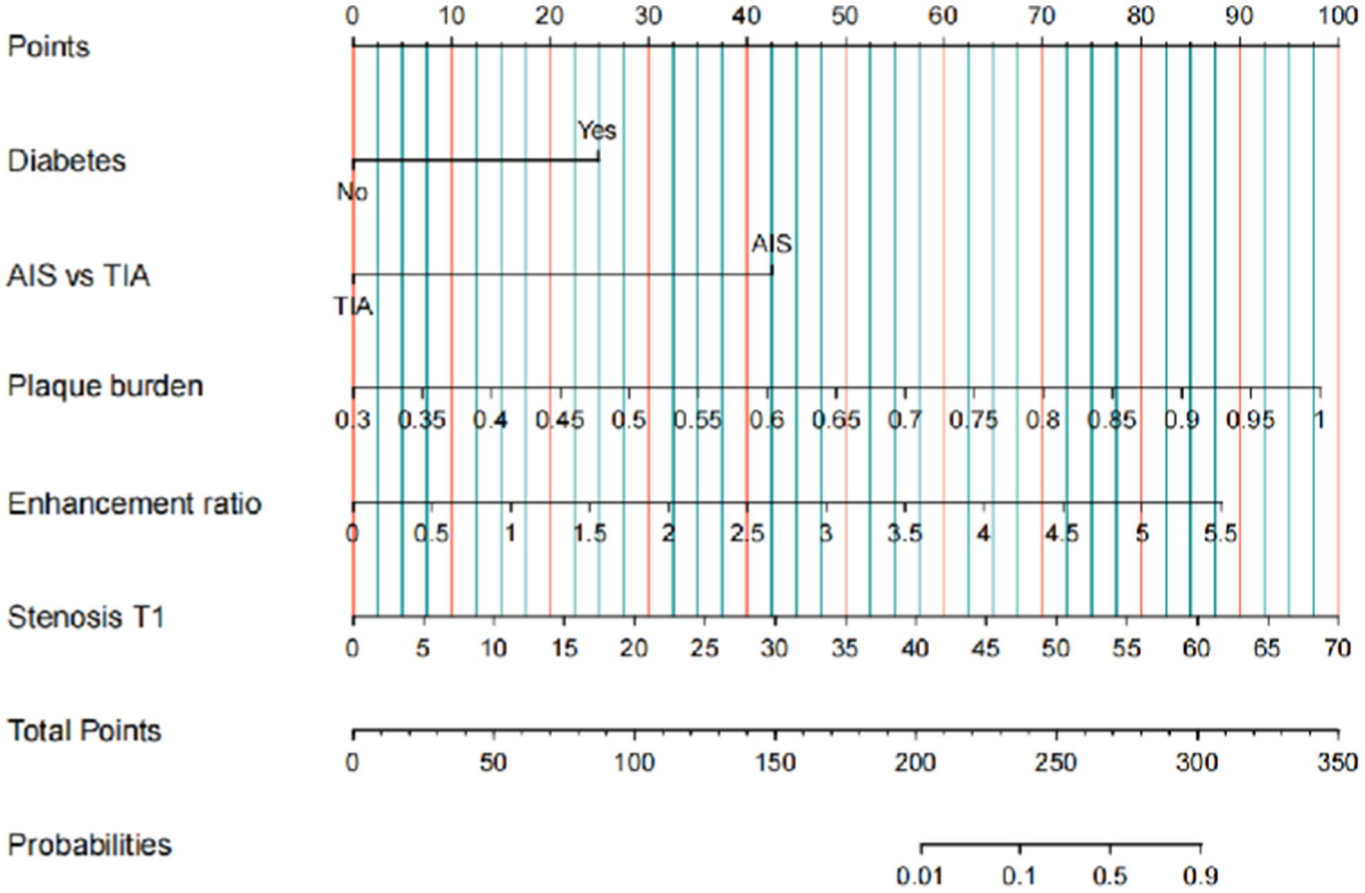
Nomogram to predict 6-month stroke recurrence of sICAS patient underwent intracranial stenting with the Neuroform Atlas Stent. Clinical factor corresponds to a specific point by drawing a line straight upward to the point axis. After the sum of the points is located on the total point axis, the sum represents the probability of a 6-month stroke recurrence rate by drawing straight down to the 6-month stroke recurrence axis. sICAS, symptomatic intracranial atherosclerotic stenosis.

A predictive model was developed based on independent predictive factors, resulting in a nomogram designed to forecast the 6-month stroke recurrence in patients with sICAS who underwent intracranial stenting using the Neuroform Atlas Stent. Each clinical factor corresponds to a specific score, and a straight line is drawn to the point axis to determine the total score, which correlates with the 6-month stroke recurrence axis and its associated probability (Figure 2).

**Figure 3:**
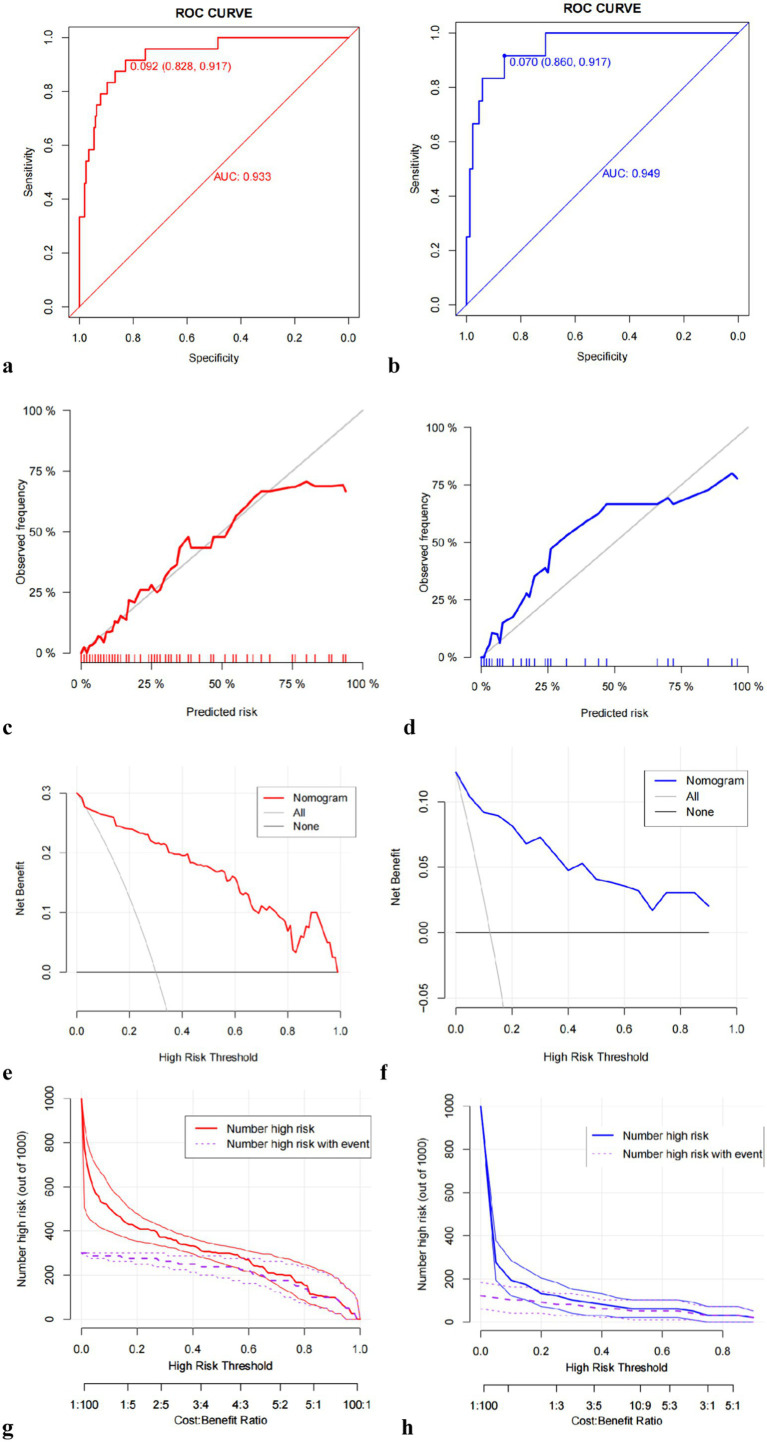
Multiple evaluation methodologies of predictive nomogram. ROC curves for predicting 6-month stroke recurrence of sICAS patients underwent intracranial stenting with the Neuroform Atlas Stent in the training set (**a**, AUC = 0.933) and validation set (**b**, AUC = 0.949); calibration curve of the predictive model showing the degree of consistency between the predicted probability and observed probability, suggesting that it is of goodness-of-fit. The gray solid line represents a perfect prediction by an ideal model, and the red and blue solid lines show the performance of the model **(c,d)**; the DCA curve for predicting 6-month stroke recurrence of sICAS patients underwent intracranial stenting with the Neuroform Atlas Stent in the training set **(e)** and validation set **(f)**; the CIC curve for predicting 6-month stroke recurrence of sICAS patients underwent intracranial stenting with the Neuroform Atlas Stent in the training set **(g)** and validation set **(h)**. ROC, receiver operating characteristic; sICAS, symptomatic intracranial atherosclerotic stenosis; AUC, area under the curve; DCA, decision curve analysis; CIC, clinical impact curve.

The identification and calibration capabilities of the model were assessed through internal validation. The receiver operating characteristic (ROC) curve analysis revealed an AUC of 0.933 for the training set and 0.949 for the validation set (Figures 3a,b), indicating strong predictive performance for 6-month stroke recurrence. The calibration curve confirmed a good fit between predicted and observed probabilities (Figures 3c,d). DCA and clinical impact curve (CIC) validated the clinical effectiveness of the nomogram. The DCA demonstrated that the net benefit of the predictive model was significantly higher than the extremes of complete intervention (All) and no intervention at all (None) in both the training and validation sets (Figures 3e,f). The CIC results showed the number of people classified as high risk by the predictive model at each threshold probability compared to the true situation, suggesting good clinical applicability (Figures 3g,h).

## 4 Discussion

### 4.1 Efficacy and safety of intracranial stenting using a Neuroform Atlas Stent

Intracranial stenting utilizing the Neuroform Atlas Stent is a potentially safe and effective approach for treating underlying stenosis and alleviating ischemic symptoms in patients with sICAS who do not respond to intensive medical treatment (10, 11). The proportion of patients with an mRS score of 0–2 was significantly higher after than before intracranial stenting, with the paired-samples *t*-test showing a statistically significant difference between mRS-pre and mRS-post (*p* = 0.027). There was a statistically significant difference in the target vessel stenosis rate at T1 compared with T0 (*p* < 0.01) as well as at T2 compared with T0 (*p* < 0.01). These results strongly suggest that intracranial stenting with the Neuroform Atlas Stent for the treatment of sICAS can effectively improve the underlying stenosis and alleviate ischemic symptoms (10, 11, 24). In addition, the statistically significant difference between T1 and T2 (*p* < 0.01) implies that this stenting approach may offer advantages for patients in both acute and chronic stages, owing to the self-expanding characteristics facilitated by the radial support forces of the Neuroform Atlas Stent (10).

Three patients experienced postoperative complications related to cerebral hemorrhage, attributed to MCA stenosis. This condition was primarily linked to hyperperfusion hemorrhage occurring in the area of previous cerebral infarction. Moreover, five patients developed postoperative complications involving cerebral infarction related to BA or VA stenosis, representing new-onset cerebral infarcts from altered blood flow or occlusion of penetrating arteries, possibly due to vascular dissection. With proper medication and rehabilitation, these five patients nearly returned to their pre-surgical state during the perioperative period and did not suffer from significant long-term effects. Our findings highlight the importance of maintaining a stable blood pressure (not exceeding 140 mmHg) in patients with anterior circulation target arteries before, during, and after surgery to mitigate complications associated with intraoperative or postoperative For patients with VA or BA as the target vessel, surgeons must ensure that the guidewire remains within the true lumen of the artery during stent implantation to minimize the risk of vascular injury or postoperative complications, such as cerebral infarction, resulting from “branch-penetrating events.” Additionally, adequate fluid resuscitation should be administered during and after the procedure to reduce the risk of complications, including cerebral infarction, associated with cerebral hypoperfusion.

### 4.2 Risk factors for stroke recurrence

The present study found that 36 of 326 patients (11.04%) experienced a recurrent stroke linked to the target artery within 6 months of intracranial stenting and no patients died. The 6-month stroke recurrence or death rate appears to be lower than 14.7% (30-day follow-up) and 19.7% (1-year follow-up) in SAMMPRIS (13) and higher than 8.5% in the WOVEN trial (1-year follow-up) (9), 10.4% for patients with drug-eluting stents, and 5.5% for patients with bare-metal stents in the study by Si et al. (25) (1-year follow-up). Univariate analysis results revealed that diabetes, current smoker, current drinker, AIS, plaque burden, enhancement ratio, and stenosis (T1) were the main factors associated with postoperative stroke recurrence in sICAS patients undergoing intracranial stenting with the Neuroform Atlas Stent. We summarize the possible mechanisms through extensive literature study as follows: (1) Diabetes: Our study identified 32 stroke recurrences in patients with diabetes compared with four among non-diabetic patients. Previous studies have established that diabetes mellitus can predict the likelihood of stroke recurrence in ICAS when considered alongside other indicators. Our results corroborate these findings, suggesting that diabetes may serve as an independent risk factor for stroke recurrence in sICAS patients undergoing intracranial stenting with the Neuroform Atlas Stent. Consequently, it is crucial to consider diabetes management in the treatment and recovery of stroke patients to mitigate the severe consequences of recurrence. (2) Current smoking: Smoking is recognized as a significant risk factor for IS (26), likely due to its immediate effects on thrombus formation in atherosclerotic arteries and its long-term effects on the development of atherosclerotic stenosis (27, 28, 29). Our findings indicate that the rate of postoperative stroke recurrence was lower among non-smokers, whereas it was elevated in current smokers. This suggests that the adverse effects of smoking on sICAS persist even after intracranial stenting, highlighting the importance of advising patients to cease smoking post-procedure. (3) Alcohol consumption: Alcohol is an important modifiable lifestyle risk factor for stroke. Several potential mechanisms may explain the link between alcohol consumption and stroke (30). Most studies suggest that light-to-moderate drinking (up to two drinks daily) reduces the risk of IS, whereas heavy drinking increases the risk of all stroke types, including hemorrhagic and ischemic stroke (31, 32). Excessive alcohol consumption can lead to high blood pressure and cerebral vasospasm, which are significant contributors to IS (33, 34). (4) AIS: Previous studies have shown patients hospitalized for AIS have a higher risk of stroke recurrence than those admitted for TIA (15). This observation aligns with our findings in patients who experienced stroke recurrence after intracranial stenting. Our data showed that the stroke recurrence rate was 14.84% (34/229) in patients with AIS and 2.06% (2/97) in patients with TIA. (5) Plaque burden on vessel wall MRI: VW-HRMRI has been recognized as a reliable and effective method for identifying and characterizing atherosclerotic lesions (35). A greater plaque burden in the cerebral artery, detected by VW-HRMRI, is independently associated with recurrent IS (36). The present study found that the plaque burden in the vessel wall was an independent risk factor for postoperative stroke recurrence in ICAS patients, even after undergoing intracranial stenting with the Neuroform Atlas Stent. This may be because plaque burden uniquely reflects both the extent of luminal narrowing and arterial remodeling (37). (6) Enhancement ratio of plaque on vessel wall MRI: Plaque enhancement on vessel wall MRI is a characteristic of vulnerable plaque, signifying active plaque inflammation, neovascularization, and vascular endothelial permeability, which have been linked to symptomatic culprit plaques (38). The probability that an intracranial atherosclerotic plaque has contributed to a recent ischemic event correlates with its contrast enhancement. It has been demonstrated that extracranial plaque enhancement is associated with inflammation and plaque disruption (39). This feature may serve as a marker for intracranial plaque instability, providing insight into the risk of stroke and emphasizing the use of three-dimensional contrast-enhanced MRI in the assessment of intracranial atherosclerotic disease. (7) Stenosis rate (T1): Our findings reveal that despite the effective deployment of the Neuroform Atlas Stent, the stenosis rate of the affected artery after the procedure (T1) was strongly associated with stroke recurrence at 6 months postoperatively. This observation aligns with the results of other studies involving pharmacological treatments for patients with sICAS. Residual stenosis rates, or hemodynamic intracranial stenosis, may respond poorly to pharmacologic therapy (14), potentially due to the unresolved hemodynamic lesions that compromise brain perfusion under conditions of reduced cerebral perfusion pressure, causing watershed infarcts (40).

### 4.3 Predictive nomogram for stroke recurrence

The incidence of stroke recurrence exhibits considerable variability, even among patients sharing identical risk factors. In cases of postoperative stroke recurrence attributed to multiple causes, analyses focusing on single factors often neglect other critical influencing elements, resulting in an inaccurate evaluation of patient prognosis. A nomogram is a valuable tool for estimating the likelihood of patient survival. It synthesizes the effects of various factors on survival rates and has been widely employed in assessing cancer patient outcomes, gradually replacing traditional prediction models. However, sICAS patients treated with the Neuroform Atlas Stent are rarely assessed using this prognostic model. Recently, Tang et al. developed a radiomics nomogram for predicting stroke recurrence in sICAS, demonstrating strong efficacy in estimating long-term remission probability (21). However, the model is based on a cohort of 156 patients with ICAS who were treated with standard antithrombotic therapy, statins, and secondary prevention (such as control of hypertension and diabetes mellitus). Intracranial stenting with the Neuroform Atlas Stent is a novel approach for treating patients with sICAS who do not adequately respond to intensive medical care. Thus, the model by Tang et al. cannot be applied to sICAS patients treated with this stenting technique. In light of technological advancements and evolving treatment paradigms, it is imperative to develop updated predictors to establish a new model specifically for intracranial stenting in the management of sICAS.

The present study for the time utilized data from 326 patients to construct a nomogram model based on intracranial stenting with the Neuroform Atlas Stent for sICAS. The accuracy of this model stems from a large patient cohort and recent advancements in sICAS. Five key factors, namely, diabetes, AIS, plaque burden on vessel wall MRI, enhancement ratio on vessel wall MRI, and stenosis (T1), were identified via multivariate logistic regression and incorporated into the nomogram scoring system. Our findings further substantiate the notion that patients without diabetes, those experiencing qualifying ischemic events such as TIA, and individuals exhibiting lower plaque burden, enhancement ratio, and stenosis rate (T1) tend to have a more favorable prognosis (21, 41). The model demonstrates robust predictive capabilities, with ROC curve analysis revealing an AUC of 0.933 for the training set and 0.949 for the validation set in predicting 6-month postoperative stroke recurrence. The calibration curve indicates a strong correlation between predicted and observed probabilities, confirming its reliability. The clinical utility of the nomogram was further validated through DCA and CIC.

## 5 Study limitations

However, the study still has several shortcomings. First, the study acknowledges that its findings are based on a specific patient population and two particular hospitals, which might limit the generalizability of the results. In future further research, we will adopt more centers’ cooperation and randomly select sICAS patients from other centers as external validation. Second, this paper is a retrospective cohort study, and the bias generated during the follow-up is unavoidable. Third, the 6-month outcomes for recurrent stroke are rather short term, and a study of the long-term risk of stroke recurrence is needed in the near future.

## 6 Conclusion

In summary, this study has demonstrated that intracranial stenting with the Neuroform Atlas Stent is a safe and effective option for treating sICAS. Diabetes, current smoker, current drinker, AIS, plaque burden on vessel wall MRI, enhancement ratio on vessel wall MRI, and stenosis (T1) are the key factors affecting the likelihood of postoperative stroke recurrence in sICAS patients undergoing this procedure.

## Data Availability

The raw data supporting the conclusions of this article will be made available by the authors, without undue reservation.
